# Mild hypothermia regulates neuronal inflammation and oxidative stress through HSP70 to alleviate brachial plexus injury

**DOI:** 10.1016/j.ibneur.2025.08.001

**Published:** 2025-08-05

**Authors:** Ke Lin, Xuhong Zhu, Jing Bai, Qi Fan, Yong Yuan, Wei Yuan, Gaoping Song

**Affiliations:** a Operating Room, The Second Affiliated Hospital of Kunming Medical University, Kunming, Yunnan 650101, China; b Department of Nursing Management, The Second Affiliated Hospital of Kunming Medical University, Kunming, Yunnan 650101, China; c Trauma Center, The Second Affiliated Hospital of Kunming Medical University, Kunming, Yunnan 650101, China

**Keywords:** Brachial plexus injury, Mild hypothermia, HSP70, Inflammation, Oxidative stress

## Abstract

**Objective:**

To investigate the function of mild hypothermia (MH) in brachial plexus injury (BPI) by regulating the 70 kDa heat shock protein (HSP70).

**Methods:**

A BPI model mouse was established to investigate the mechanism of MH and HSP70 on BPI through hematoxylin-eosin staining, enzyme-linked immunosorbent assay (ELISA), dichloro-dihydro-fluorescein diacetate (DCFH-DA) staining, and western blotting assays. A cellular model was established by stimulating the motor neuron-like cell line NSC-34 cells with lipopolysaccharide (LPS). The effects of MH and HSP70 on LPS-induced NSC-34 cell proliferation, cytokines, apoptosis, and oxidative stress were studied using western blotting, cell counting kit-8, ELISA, and DCFH-DA staining.

**Results:**

MH treatment inhibited the expression of HSP70 in the brachial plexus tissues of BPI mice, reduced levels of pro-inflammatory cytokines and oxidative stress, and diminished the apoptosis in neural tissues. Knockdown of HSP70 further promoted the protective effects of MH on BPI mice. Cell experiments indicated that MH treatment alleviated the inhibitory effect of LPS on the proliferation of NSC-34 cells by inhibiting HSP70 protein expression, while also reducing reactive oxygen species, oxidative stress, and apoptosis rates.

**Conclusions:**

MH has protective effects on BPI mice by downregulating HSP70 level, inhibiting cellular oxidative stress and apoptosis.

## Introduction

The brachial plexus is composed of C5-C8 cervical nerves and T1 thoracic nerve roots, which innervate the motor and sensory functions of the upper limb and scapular muscles. Brachial plexus injury (BPI) is a common peripheral nerve injury, which is mostly caused by motor vehicle accidents or accidental falls. The patient’s upper limb function is partially or completely lost, which seriously affects the quality of life of patients (Gutkowska et al., 2020). The diagnosis of BPI includes clinical evaluation, imaging examination, and electrophysiological testing (Portwood et al., 2022). The main clinical treatment methods for BPI include neurotrophic drug therapy, surgery combined with routine postoperative rehabilitation therapy, etc (Gutkowska et al., 2020). There is a significant difference in the cure rate among patients with different degrees of injury (Midha, 1997). Age and duration of injury are also important factors affecting healing (Gutkowska et al., 2020).

Mild hypothermia (32–35 ℃, MH) is one of the important treatments for neurological diseases, which can improve neurological function by reducing local metabolism, minimizing the production of harmful substances, and blocking the persistent nerve damage effects caused by secondary injury factors after injury. MH has been reported to have good therapeutic effects in neonatal brain injury, nerve injury, and cardiovascular diseases. A meta-analysis by Huang et al., (2020)showed that MH therapy can improve the long-term recovery of neurological function in patients with severe traumatic brain injury. MH activated the Nrf2-ARE pathway, inhibited oxidative stress levels, and have neuroprotective effects against traumatic brain injury (Yan et al., 2022). MH reduced the hypoxic-ischemic injury of axons, promoted the myelination of oligodendrocytes and the proliferation of early oligodendrocyte progenitors, and had a protective effect on nerve injury in neonatal rats (Xiong et al., 2013). Treatment with MH at 33 ℃ enhanced vitality and inhibit cell apoptosis in oligodendroglioma cells exposed to peroxides, and reduced mitochondrial oxidative stress (Liang et al., 2023). Jiao et al. (2024) treated acute ischemic stroke patients with thrombolytic therapy with MH combined with distal ischemic postconditioning, and found that this treatment has a positive effect on brain protection and reduces oxidative stress and inflammatory reaction. However, the role of MH in BPI remains clear.

The 70 kDa heat shock protein (HSP70) is a molecular chaperone protein, which is a heat stress protein widely existing in animals. It can correct protein’s misfolding and maintain cell homeostasis. HSP70 is highly conserved in the process of biological evolution and participates in important physiological functions such as maintaining muscle homeostasis and growth, cell protection, cell survival, and cell immunity (Yang et al., 2024). Vishwakarma et al., (2017) reported that MH reduces the expression of HSP70, improves the structure and function of undifferentiated human neural precursor cells and differentiated neurons, and has a protective effect against traumatic brain injury. Sevoflurane exerts neuroprotective effects on rats with cerebral ischemia-reperfusion injury by reducing the expression of HSP70 (Yu et al., 2020). However, the function of HSP70 in BPI is still unclear.

In the present study, we described the effects of MH treatment on BPI mouse and cell models. Results found that MH promoted the proliferation in lipopolysaccharide (LPS)-stimulated NSC-34 cells, reduced the production of cytokines, and decreased damage in BPI mice. Mechanism studies indicated that MH has protective effects on BPI mice by reducing the expression of HSP70.

## Methods

### Experimental animals

The 6–8 weeks female C57BL/6 mice (20–25 g) were provided by Hunan SiLaiKe JingDa Experimental Animal Co., Ltd. Mice were kept in a specific pathogen free environment with room temperature of 23–25 ℃ and humidity of 55 %–60 %, and they ad libitum access to food and water. This study was carried out in accordance with the National Institute of Health Guide for the Care and Use of Laboratory Animals (NIH Publications No. 80–23) revised 1996 and approved by the Experimental Animal Ethics Committee of Yunnan Labreal Biotechnology Co., Ltd (PZ20231217). Mice were randomly divided into sham, BPI, BPI+MH, BPI+MH+LV-NC, and BPI+MH+LV-HSP70 group, n = 6.

### Establishment of the BPI mice model

Pentobarbital sodium (1 %) was used to anesthetize mice. A transverse incision of 0.8–1.2 cm was made 0.3–0.5 cm below the clavicle of the left upper limb. The mouse brachial plexus was isolated and C7 nerve roots were exposed. The C7 nerve was wrapped with a 2–0 silk thread and avulsed at a constant speed through the stretch of the thread. After avulsion, the stump was put back into the muscle space between pectoralis major and musculus minor, and the wound was sutured with 3–0 silk thread. The sham group mice received the same exposure of the brachial plexus nerve, but without any damage. The BPI+MH+LV-NC (knockdown of NC, KD-NC) group and BPI+MH+LV-HSP70 (knockdown of HSP70, KD-HSP70) group mice were injected with 100 μL of lentivirus (LV, Shanghai Genomeditech) through the tail vein 5 days before BPI, while the other groups of mice were injected with an equal amount of physiological saline.

### Treatment with MH

Within 5 min postoperatively, mice in the MH group received localized cooling at the BPI site using an ice pack to maintain tissue temperature 32 ℃–34 ℃ for 2 h, while rectal temperature was maintained at 36–37 ℃. Both rectal and injured brachial plexus temperature were monitored with an electronic thermometer and recorded at 10-minute intervals. MH therapy was administered on postoperative days 1, 3, 7, 14, 21, 28, 35, and 42.

### Terzis grooming test (TGT) score

TGT was used to assess the motor function of mouse upper limbs. Bacteria-free water was sprayed on the neck of mice with a 20 mL syringe to induce the combing behavior of bilateral upper limbs. The function of the right upper limb was evaluated according to the following 0–5 scale: 0 grade, the affected upper limb did not respond; Grade 1, the elbow of the affected side can be bent, but the upper limb of the affected side can’t touch the nose; Grade 2, the affected upper limb can touch the nose; Grade 3, the affected elbow can be bent, and the affected forelimb can touch the lower part of the eye; Grade 4, the affected forelimbs can contact the eyes; Grade 5, the affected forelimb can touch the ear or behind the ear.

### Hematoxylin-eosin (HE) staining

The brachial plexus of mice was fixed with 4 % paraformaldehyde, embedded in paraffin and sliced. The slices were baked in the oven and treated with xylene and gradient ethanol. The slices were rinsed with distilled water, stained with hematoxylin (Sigma-Aldrich, St. Louis, MO, USA) and differentiated with 1 % hydrochloric acid and ethanol. After ethanol dehydration, the slices were stained with 0.5 % eosin (Sigma-Aldrich, St. Louis, MO, USA). Slices were treated with anhydrous ethanol and xylene, sealed, and observed with a random field under a microscope.

### Enzyme-linked immunosorbent assay (ELISA)

TNF-α, IL-1β, and IL-6 ELISA kits (Shanghai Beyotime, China) were used to detect the concentrations of TNF-α, IL-1β, and IL-6. Briefly, prepare different concentrations of standards according to the kit instructions. The samples and standards with different concentrations were added into the corresponding wells of 96-well plates, and 100 μL of biotinylated antibody was added into each well. The 96-well plates were blocked and incubated at 25 ℃ for 1 h. Streptavidin labeled with horseradish peroxidase (100 μL) was added to each well and incubated at 25 ℃ for 20 min. After treating with TMB reagent, the optical density at 450 nm was detected using a microplate reader.

### Dichloro-dihydro-fluorescein diacetate (DCFH-DA)

Mouse brachial plexus nerve tissues were prepared as a single cell suspension. DCFH-DA probe (Sigma-Aldrich, St. Louis, MO, USA) was added to the tissue samples or NSC-34 cell samples, with a final concentration of 10 μmol/L. The cells were incubated at 37 ℃ for 30 min. The fluorescence intensity was measured by flow cytometry.

### Western blotting

Mouse brachial plexus nerve tissue and cells were thoroughly lysed using RIPA for total protein extraction. The concentrations of the protein were detected using a spectrophotometer. Protein samples were transferred to polyvinylidene fluoride membrane after polyacrylamide gel electrophoresis, and 5 % skim milk was used to seal membranes. HSP70, B-cell lymphoma 2 (Bcl-2), Bcl2-associated X (Bax), Cleaved-caspase 3 (Abcam, Shanghai), malondialdehyde (MDA) (Amyjet Scientific, Wuhan), and superoxide dismutase (SOD) (Beijing Bai ao lai bo science and technology co., ltd) primary antibodies were incubated overnight with the membranes at 4 ℃. After rinsing the membranes, secondary antibodies were added and incubated with the membranes for 2 h at 25 ℃. Enhanced chemiluminescence reagent was used to visualize the proteins, and Image J software was used to analyze the gray value of proteins.

### Cell culture

NSC-34 cells were provided by Shanghai YaJi Biotechnology Co., Ltd. (CAT#: YS1827C). Cells were seeded in DMEM medium supplemented with 10 % fetal bovine serum, and placed in a cell incubator with 37 ℃ and 5 % CO_2_.

### Cell treatment

NC group cells were cultured normally. LPS group cells were treated with 100 ng/mL LPS for 24 h. The LPS+MH group cells were induced with MH at 32 ℃ and treated with 100 ng/mL LPS for 24 h. The sh-NC, sh-HSP70, pcDNA-NC, and pcDNA-HSP70 groups of cells were transfected with sh-NC, sh-HSP70, pcDNA-NC, and pcDNA-HSP70 for 48 h, followed by LPS and MH treatment.

### Cell counting kit-8 (CCK-8)

The transfected cells were digested with 0.25 % trypsin to obtain a cell suspension. Cells (5000 cell/well) were seeded into a 96 well plate and cultured the cells until the density reaches around 75 %. After treatment with MH and LPS, 10 μL of CCK-8 reagent (MedChem Express, Monmouth Junction, NJ, USA) was added to each well, and the cells were cultured at 37 ℃ for 3 h. The optical density at 450 nm was measured using a microplate analyzer.

## Statistical analysis

GraphPad Prism 8.0 software was used to analyze the data. One-way analysis of variance was used to analyze the differences among multiple groups, and student’s t test was used to analyze the differences between the two groups. Experimental data were expressed as “mean ± standard deviation”. P value of less than 0.05 was considered statistically significant.

## Results

### MH alleviates BPI in mice

**Fig. 1:**
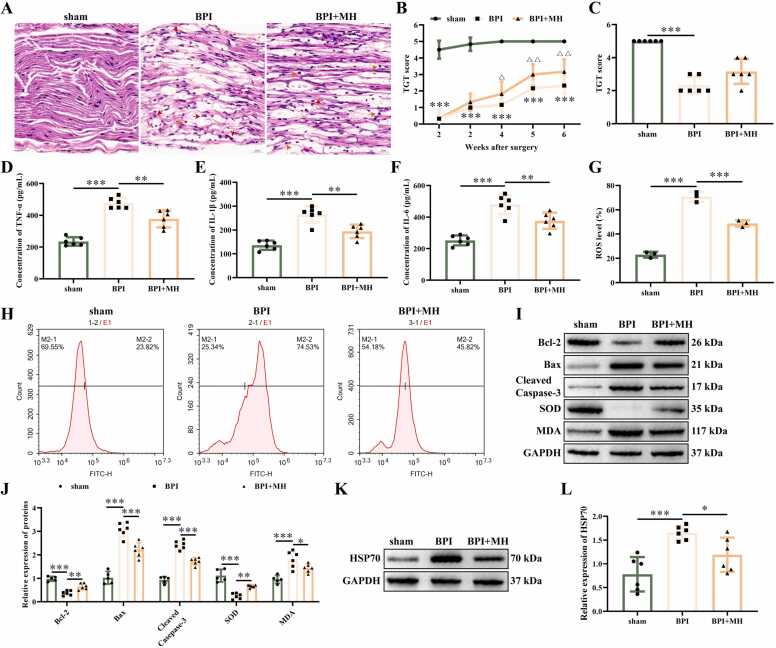
MH alleviates BPI in mice. (A) Representative images of HE in the BPI of mice. Scare bar: 20 μm. Red arrow: neutrophils; Blue arrow: macrophage; Yellow arrow: lymphocytes. (B) TGT score of mice at different time points after surgery. (C) TGT score of mice on the 14th day after surgery. (D) The concentration of TNF-α detected by ELISA. (E) The concentration of IL-1β detected by ELISA. (F) The concentration of IL-6 detected by ELISA. (G) The percentage of ROS. (H) Representative images of ROS detected by flow cytometry. (I and J) The protein gel images and relative expression statistics of Bcl-2, Bax, Cleaved caspase-3, SOD, and MDA. (K-L) The protein gel images and relative expression statistics of HSP70. *P < 0.05, * *P < 0.01, ***P < 0.001. *n* = 6. (For interpretation of the references to color in this figure legend, the reader is referred to the web version of this article.)

MH was used to treat BPI model mice. HE staining found that in the sham group, cellular morphology remained normal, with intact cell bodies and clearly visible nucleoli (Fig. 1A). In BPI group, tissues exhibited uneven atrophy accompanied by localized sparsity, nerve fibers disruption, and altered morphology in the majority of cells. Compared with the BPI group, MH treatment group showed significant improvement in cellular morphology and a substantial reduction in axonal disruption. TGT score of mice in BPI group increased gradually with the increase of time from 2 to 6 weeks, and then stabilized (Fig. 1B). At the sixth week, the TGT value was 2.33 (Fig. 1C). The TGT score of MH treatment group was higher than that of the BPI group, with a TGT score of 3.167 at the sixth week. ELISA, flow cytometry, and western blotting assays revealed that compared with sham group, the concentration of TNF-α (Fig. 1D), IL-1β (Fig. 1E), IL-6 (Fig. 1F), the percentage of reactive oxygen species (ROS) (Fig. 1G-H), and the proteins of Bax, Cleaved-caspase 3, and MDA (Fig. 1I-J) were increased. The expression of Bcl-2, and SOD in BPI group decreased in comparison with sham group (Fig. 1I-J). After treating with MH, the concentration of TNF-α, IL-1β, and IL-6 and the percentage of ROS decreased. The levels of Bcl-2, and SOD were promoted in MH treated group, Bax, Cleaved-caspase 3, and MDA were inhibited (Fig. 1D-J). Western blotting was used to detect the expression of HSP70, and found that HSP70 was upregulated in BPI group in comparison with sham group (Fig. 1K-L), and inhibited in MH treated group. These data indicates that treatment with MH treatment inhibits inflammatory cytokines and oxidative stress in mouse nerve tissue and alleviates BPI.

### MH regulates LPS-induced inflammation and oxidative damage in NSC-34 cells

**Fig. 2:**
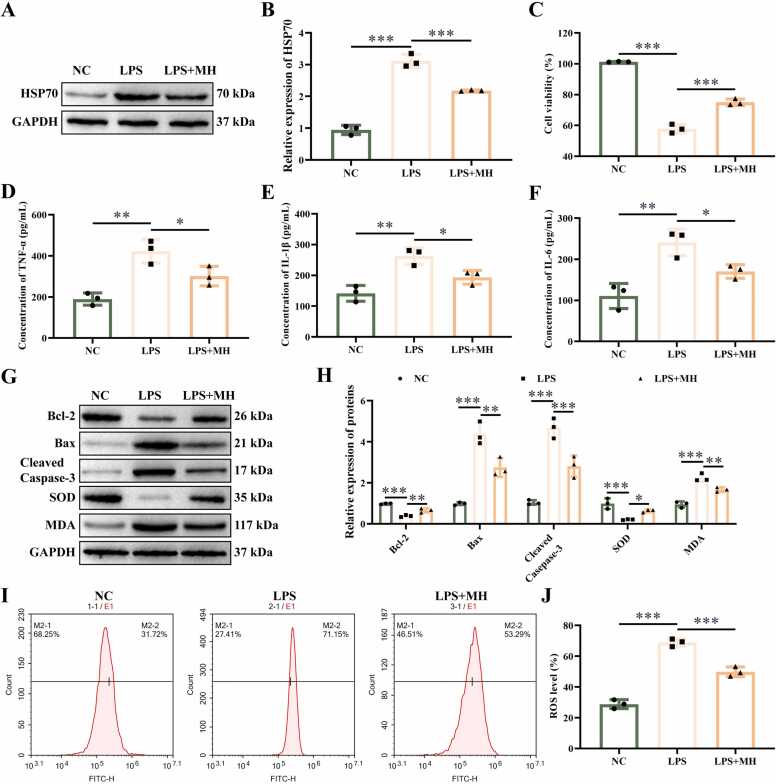
MH regulates LPS-induced inflammation and oxidative damage in NSC-34 cells. (A-B) The protein gel images and relative expression statistics of HSP70. (C) Cell viability was detected using CCK-8 assay. (D) The concentration of TNF-α detected by ELISA. (E) The concentration of IL-1β detected by ELISA. (F) The concentration of IL-6 detected by ELISA. (G-H) The protein gel images and relative expression statistics of Bcl-2, Bax, Cleaved caspase-3, SOD, and MDA. (I) The percentage of ROS. (J) Representative images of ROS detected by flow cytometry. *P < 0.05, **P < 0.01, ***P < 0.001. *n* = 3.

Next, we explore the role of MH in LPS-induced NSC-34 cells. NSC-34 cells were treated with LPS and MH. HSP70 level was increased in LPS-treated NSC-34 cells, and inhibited by treating with MH (Fig. 2A-B). The cell viability of NSC-34 cells NSC-34 cells in LPS treatment group was inhibited, and MH treatment could increase the cell activity (Fig. 2C). Treatment with LPS promoted the concentrations of TNF-α (Fig. 2D), IL-1β (Fig. 2E), and IL-6 (Fig. 2F), inhibited the levels of Bcl-2, and SOD (Fig. 2G-H), enhanced the proteins of Bax, Cleaved-Caspase-3, and MDA and upregulated the percentage of ROS (Fig. 2I-J). Compared with LPS-induced group, treatment with MH inhibited the cell viability (Fig. 2C) and concentrations of cytokines (Fig. 2D-F), decreased cell apoptosis rate (Fig. 2G-H) and the level of oxidative stress in NSC-34 cells (Fig. 2G-J). These data suggest that treatment with MH promotes cell viability and inhibits inflammation and oxidative stress in LPS treated NSC-34 cells through upregulating HSP70 level.

### MH potentiates the effects of HSP70 knockdown on LPS-induced inflammatory response and oxidative damage in NSC-34 cells

**Fig. 3:**
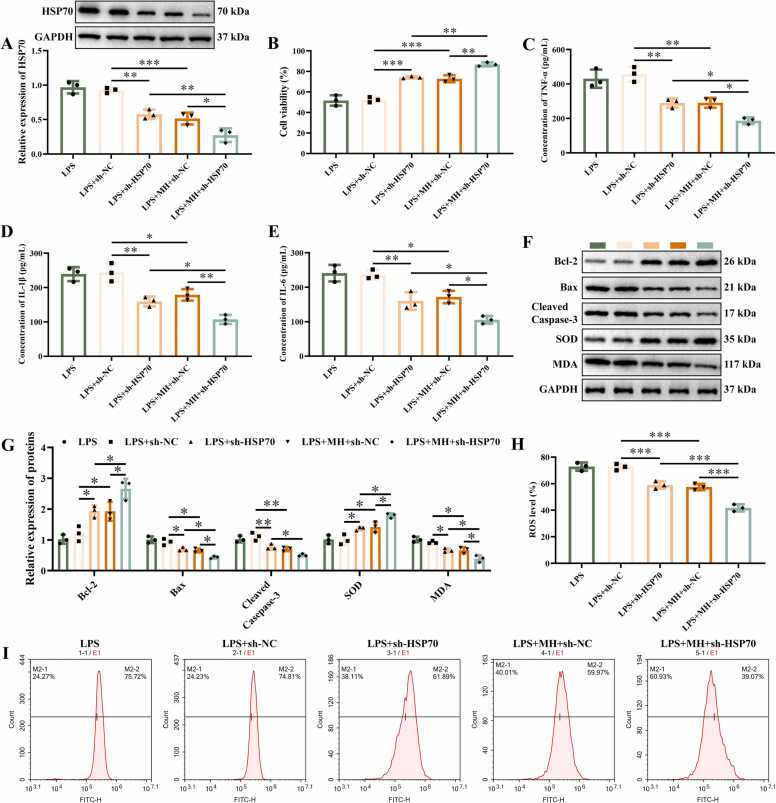
MH potentiates the effects of HSP70 knockdown on LPS-induced inflammatory response and oxidative damage in NSC-34 cells, (A) The protein gel images and relative expression statistics of HSP70. (B) Cell viability was detected using CCK-8 assay. (C) The concentration of TNF-α detected by ELISA. (D) The concentration of IL-1β detected by ELISA. (E) The concentration of IL-6 detected by ELISA. (F-G) The protein gel images and relative expression statistics of Bcl-2, Bax, Cleaved caspase-3, SOD, and MDA. (H) The percentage of ROS. (I) Representative images of ROS detected by flow cytometry. *P < 0.05, **P < 0.01, ***P < 0.001. *n* = 3.

We transfected sh-NC and sh-HSP70 into NSC-34 cells. Western blotting results revealed that transfection with sh-HSP70 significantly inhibited the expression of HSP70 (Fig. 3A-B). Treatment with MH repressed the expression of HSP70. CCK-8, ELISA, Western blotting, and flow cytometry experiments indicated that compared with LPS+sh-NC group, knockdown of HSP70 enhanced cell viability, inhibited cytokines TNF-α (Fig. 3C), IL-1β (Fig. 3D), and IL-6 (Fig. 3E), promoted the proteins of Bcl-2, and SOD (Fig. 3F-G), repressed Bax, Cleaved-Caspase-3, and MDA protein. Percentage of ROS was inhibited by inhibiting of HSP70 (Fig. 3H-I). Compared with LPS+MH+sh-NC group, knocking down HSP70 got the same result as before (Fig. 3B-I). These results indicate that HSP70 inhibition enhances cell viability, suppresses cytokine release and oxidative stress, and reduces apoptosis in LPS-induced NSC-34 cells. Moreover, MH treatment potentiates the restorative effects of HSP70 knockdown on LPS-injured NSC-34 cells.

### MH attenuates the effects of HSP70 overexpression on LPS-induced inflammatory response and oxidative damage in NSC-34 cells

**Fig. 4:**
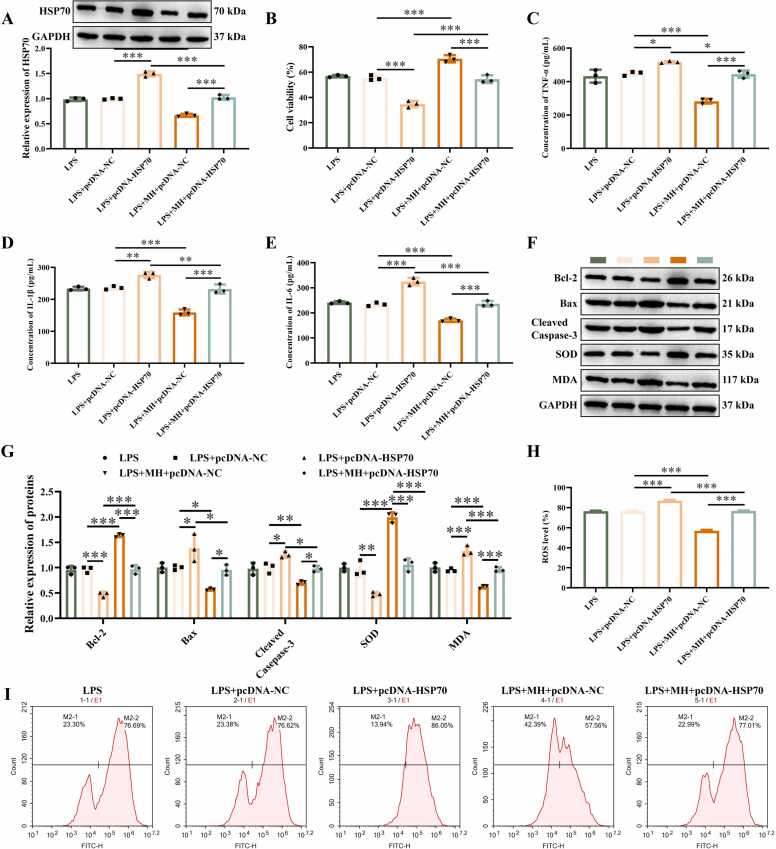
MH attenuates the effects of HSP70 overexpression on LPS-induced inflammatory response and oxidative damage in NSC-34 cells, (A) Detection of HSP70 expression in NSC-34 cells transfected with pcDNA-HSP70. (B) Cell proliferation viability assessed by CCK-8 assay. (C-E) Concentrations of TNF-α, IL-1β, and IL-6 measured using ELISA kits. (F-G) Protein expression levels of Bcl-2, Bax, Cleaved caspase-3, SOD, and MDA analyzed by Western blot. (H-I) Intracellular ROS levels quantified via flow cytometry. *P < 0.05, **P < 0.01, ***P < 0.001. *n* = 3.

After transfection of pcDNA-NC and pcDNA-HSP70 into NSC-34 cells, western blotting analysis revealed that HSP70 expression showed no significant changes in the LPS+pcDNA-NC and LPS groups (Fig. 4A), whereas it was markedly elevated in the pcDNA-HSP70-transfected group. MH treatment attenuated the pcDNA-HSP70-mediated upregulation of HSP70.

Results from CCK-8 assay, ELISA, western blotting, and flow cytometry demonstrated that, compared with the LPS+pcDNA-NC group, HSP70 overexpression significantly inhibited NSC-34 cell viability (Fig. 4B), increased levels of the proinflammatory cytokines TNF-α, IL-1β, and IL-6 (Fig. 4C-E), suppressed Bcl-2 and SOD protein expression, and upregulated Bax, cleaved caspase-3, MDA (Fig. 4F-G), and ROS levels (Fig. 4H-I). In contrast, LPS+MH+pcDNA-NC treatment alleviated LPS-induced NSC-34 cell injury, reduced inflammatory cytokine levels, decreased apoptosis, and lowered ROS levels compared with the LPS+pcDNA-NC group. Furthermore, MH treatment increased cell viability and reduced proinflammatory cytokine levels, apoptosis rate, and ROS levels relative to the LPS+pcDNA-HSP70 group. These findings suggest that MH attenuates LPS-induced damage in NSC-34 cells by downregulating HSP70 expression.

### MH alleviates BPI in mice by regulating HSP70

**Fig. 5:**
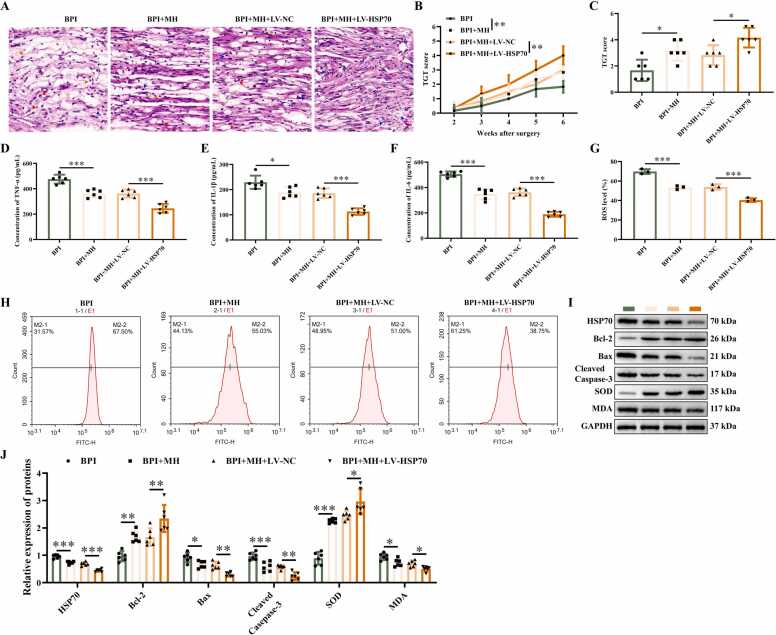
MH alleviates BPI in mice through HSP70, (A) Representative images of HE in the BPI of mice. Scare bar: 20 μm. Red arrow: neutrophils; Blue arrow: macrophage; Yellow arrow: lymphocytes. (B) TGT score of mice at different time points after surgery. (C) TGT score of mice on the 14th day after surgery. (D) The concentration of TNF-α detected by ELISA. (E) The concentration of IL-1β detected by ELISA. (F) The concentration of IL-6 detected by ELISA. (G) The percentage of ROS. (H) Representative images of ROS detected by flow cytometry. (I-J) The protein gel images and relative expression statistics of HSP70, Bcl-2, Bax, Cleaved caspase-3, SOD, and MDA. *P < 0.05, **P < 0.01, ***P < 0.001. *n* = 6. (For interpretation of the references to color in this figure legend, the reader is referred to the web version of this article.)

LV vectors carrying sh-NC and sh-HSP70 were injected into mice to explore the function of HSP70 in BPI. HE staining revealed inflammatory cells infiltration, partial nerve fiber disruption, and disorganized cellular arrangement in the neural tissues of BPI group mice (Fig. 5A). Following MH treatment, both inflammatory cell infiltration and nerve fiber disruption were reduced. Compared with the MH treatment group, the HSP70-knockdown MH treatment group exhibited further alleviation of tissue damage. The TGT score of MH treated mice increased in comparison with BPI group (Fig. 5B-C), and there was no significant difference between LV-NC injected mice and MH treated mice. The TGT score of HSP70inhibition mice was higher than that of the control group. ELISA and flow cytometry assays revealed that inhibition of HSP70 repressed the concentration of TNF-α (Fig. 5D), IL-1β (Fig. 5E), and IL-6 (Fig. 5F), decreased the percentage of ROS (Fig. 5G-H). Western blotting results found that the levels of HSP70, Bax, Cleaved-Caepase-3, and MDA proteins were inhibited by knocking down HSP70 (Fig. 5I-J). The levels of Bcl-2, and SOD proteins increased in HSP70 inhibition group. These findings suggest that treatment with MH inhibits the product of cytokines and ROS, decreases oxidative stress, and alleviates the injury in BPI mice through inhibiting HSP70 expression.

## Discussion

BPI is one of the peripheral nerve injury diseases characterized by partial or complete loss of sensory and motor functions of upper limbs. According to the degree of injury, BPI can be divided into nerve amputation, nerve apraxia, and axonal amputation, which has a high disability rate and seriously affects the quality of life of patients. The acute phase oxidative damage and inflammatory response caused by BPI are early pathological changes in nerve damage, which can lead to massive neuronal death. Reducing the inflammation and oxidative damage in the early injury stages and promoting nerve regeneration are key to the treatment of BPI. In this study, we found that MH inhibited the oxidative stress and inflammatory reaction in LPS-induced NSC-34 cells by regulating HSP70, thus exerting a protective role on BPI.

MH is a widely used neuroprotective strategy that effectively alleviates neurological disorders by reducing the temperature of local tissues in the body, lowering metabolic rates, and reducing the production of metabolites (Wang et al., 2024). Tsai et al., (2012) found that MH inhibits ectopic discharge, glial cell activation, and neuropathic pain in the early stages of chronic constriction injury of the median nerve. This study demonstrated that the levels of inflammatory cytokines and oxidative stress were reduced in brachial plexus nerve tissue of BPI mice treated with MH, and cell apoptosis was reduced. The results of cell experiments indicated that treatment with MH increased the proliferation of LPS-stimulated NSC-34 cells, reduced the levels of inflammatory cytokines and oxidative stress, repressed apoptosis rate and reduced HSP70 protein level. MH treatment has also been shown to have a relieving effect on other diseases. For instance, MH therapy improved myocardial ischemia-reperfusion injury by upregulating O-GlcNAcylation levels of COX10, enhanced mitochondrial function, and inhibited ROS levels (Deng et al., 2024a). MH reduced neuronal apoptosis and cell ferroptosis by activating SIRT1/AMPK signaling pathway, and obviously reversed the pathological changes of cerebral ischemia-reperfusion rats (Li et al., 2024a). MH therapy reduced ferroptosis induced by myocardial ischemia/reperfusion and protected myocardial cells by activating PI3K/AKT signaling pathway and inhibiting TRPM7 expression (Li et al., 2024b).

HSP70 is an important protein that maintains normal cellular function and homeostasis in the body. It can cooperate with molecular chaperone to maintain protein homeostasis in cells through various functions such as folding and synthesizing new proteins, increasing the solubility of protein aggregates, assisting in protein transmembrane transport, and regulating the formation and dissociation of multi protein complexes (Rosenzweig et al., 2019). In addition to participating in normal physiological activities of the body, HSP70 is involved in cancer, neurodegenerative diseases, and infectious diseases. In people with preeclamptic, the mitochondrial oxidative stress and HSP70 expression of mitochondria were increased (Padmini et al., 2009). Melatonin had neuroprotective effects on chronic cerebral hypoperfusion in ovariectomized rats by reducing oxidative stress and HSP70 expression (Ozacmak et al., 2009). In the present study, we found that knockdown of HSP70 in LPS-induced NSC-34 cells reduced inflammatory cytokine levels, ROS, and apoptosis. Treatment with MH and knockdown of HSP70 further promoted the protective effect of MH on BPI mice. HSP70 overexpression and HSP70 knockdown exerted opposing effects on LPS-induced NSC-34 cell injury. However, some studies have shown that HSP70 expression are increases when they are stimulated by external stimuli, alleviates cell damage, increases body resistance, and plays an important role in the process of apoptosis, oxidation, and immunity. The addition of recombinant HSP70 to the sciatic nerve transection model rats observed a decrease in spinal ganglion cell apoptosis and an increase in nerve tissue regeneration markers (Demyanenko et al., 2024). HSP70 reduced the apoptosis and oxidative stress in tert-butyl hydrogen peroxide stimulated PC12 cells by activating Nrf2/HO1 signaling pathway (Deng et al., 2024b). Knockdown of HSP70 can promote ROS production, oxidative stress and apoptosis in Burkitt lymphoma Raji cells (Khan et al., 2020). Knockdown of HSP70 increased ROS production, oxidative stress, and apoptosis in Burkitt’s Lymphoma Raji Cells (Xu et al., 2018).

In conclusion, our study indicated that MH treatment protects BPI mice by inhibiting inflammatory cytokine levels, reducing ROS and oxidative stress, and attenuating apoptosis, which may be mediated by the inhibition of HSP70 expression. However, this study has several limitations. The NSC-34 cell line is a hybrid of spinal motor neurons and neuroblastoma cells, which may retain certain tumorigenic characteristics. As an immortalized cell line, NSC-34 cell exhibits partial loss of mature motor neuron functionality. Furthermore, since the brachial plexus does not contain neuronal cell bodies, the use of LPS-induced NSC-34 cell injury to model BPI has inherent limitations. This study represents only a preliminary exploration, and further validation using primary cell models is required.

## Authors contributions

**Ke Lin** conceived and designed the experiments, and wrote the original draft. **Wei Yuan** and **Gaoping Song** performed the experiments. **Jing Bai** and **Qi Fan** analyzed the data. **Yong Yuan** contributed reagents / materials / analysis tools. **Xuhong Zhu** wrote, reviewed and edited the final manuscript.

## CRediT authorship contribution statement

**Gaoping Song:** Validation, Methodology, Investigation. **Ke Lin:** Funding acquisition, Conceptualization. **Xuhong Zhu:** Writing – review & editing. **Jing Bai:** Visualization, Formal analysis, Data curation. **Qi Fan:** Visualization, Formal analysis, Data curation. **Yong Yuan:** Resources. **Wei Yuan:** Validation, Methodology, Investigation.

## Ethics

This study was carried out in accordance with the National Institute of Health Guide for the Care and Use of Laboratory Animals (NIH Publications No. 80–23) revised 1996 and approved by the Experimental Animal Ethics Committee of Yunnan Labreal Biotechnology Co., Ltd (PZ20231217).

## Funding

This study was supported by Joint Project of Yunnan Province Science and Technology Department - Kunming Medical University (202201AC070949).

## Conflicts of Interest

The authors declare that they have no conflict of interest.

## Data Availability

Data is provided within the manuscript or supplementary information files.
